# Epidemiology of tetanus in Canada, 1995–2019

**DOI:** 10.17269/s41997-022-00732-7

**Published:** 2023-01-17

**Authors:** Nicole Salem, Grace Huang, Susan G. Squires, Marina I. Salvadori, Y. Anita Li

**Affiliations:** 1 Infectious Diseases Programs Branch, Public Health Agency of Canada, Ottawa, ON Canada; 2 Department of Pediatrics, Faculty of Medicine and Health Sciences, McGill University, Montreal, QC Canada

**Keywords:** Tetanus, Canada, Epidemiology, Public health surveillance, Tétanos, Canada, épidémiologie, surveillance de la santé publique

## Abstract

**Objectives:**

This report aims to use tetanus hospitalization data to describe the epidemiology in Canada from 1995 to 2019 and to assess progress on national reduction targets, including validating that Canada has eliminated maternal and neonatal tetanus (MNT).

**Methods:**

Tetanus hospitalizations and fatalities occurring between 1995 and 2019 were retrieved from the Canadian Institute for Health Information (CIHI) and Statistics Canada. Cases coded with ICD-10 codes *A33*, *A34*, or *A35* as the primary diagnosis (or ICD-9 equivalents) were included. The Canadian national case definition was used for generic tetanus and definitions from the World Health Organization were referenced for MNT. R version 4.0.2 was used for analyses.

**Results:**

From 1995 to 2019, 155 non-MNT, 6 neonatal, and 0 maternal tetanus cases were retrieved from CIHI. However, all 6 neonatal cases were excluded after validating with provincial/territorial public health officials. In the same time period, there were 91 national notifications of tetanus. Cases were distributed relatively equally across the country, with the exception of the territories, where zero cases were reported. Adults 75 and over had significantly higher incidence rates compared to younger age groups (*p*<0.001). Ten deaths were reported during the timeframe.

**Conclusion:**

Tetanus incidence remains low and hospitalization data reveal that Canada has met its reduction target of maintaining 5 cases or fewer annually in recent years. For MNT, Canada has successfully met the elimination target of zero cases. Continued vaccination efforts must be practiced for all age groups, including those aged 75 years and older, to sustain targets moving forward.

## Résumé

**Objectifs:**

Ce rapport vise à utiliser les données sur les hospitalisations dues au tétanos pour décrire l’épidémiologie au Canada de 1995 à 2019 et pour évaluer les progrès réalisés par rapport aux objectifs nationaux de réduction, notamment pour confirmer que le Canada a éliminé le tétanos maternel et néonatal (TMN).

**Méthodes:**

Les données sur les hospitalisations et les décès attribuables au tétanos survenus entre 1995 et 2019 proviennent de l’Institut canadien d’information sur la santé (ICIS) et de Statistique Canada. Les cas pour lesquels les codes sont CIM-10 *A33*, *A34* ou *A35* comme diagnostic primaire (ou leurs équivalents CIM-9) sont inclus. La définition de cas nationale canadienne a été utilisée pour le tétanos générique et les définitions de l’Organisation mondiale de la santé ont servi de référence pour le TMN. La version 4.0.2 de R a été utilisée pour les analyses.

**Résultats:**

De 1995 à 2019, il y a eu 155 cas de tétanos autres que TMN, 6 cas de tétanos néonatal et 0 cas de tétanos maternel selon l’ICIS. Cependant, les 6 cas de tétanos néonatal ont été exclus après validation auprès des responsables de la santé publique provinciaux/territoriaux. Durant la même période, il y a eu 91 notifications nationales de cas de tétanos. Ces cas étaient répartis de manière relativement égale à travers du pays, à l’exception des territoires, où aucun cas n’a été signalé. Les adultes de 75 ans et plus présentaient des taux d’incidence significativement plus élevés que les groupes d’âge plus jeune (*p*<0,001). Dix décès ont été rapportés pendant cette période.

**Conclusion:**

L’incidence du tétanos reste faible et les données sur les hospitalisations révèlent que le Canada a atteint son objectif de réduction de 5 cas ou moins par an au cours des dernières années. En ce qui concerne le TMN, le Canada a réussi à atteindre l’objectif d’élimination des cas. Il faut poursuivre les efforts de vaccination pour tous les groupes, y compris les personnes âgées de 75 ans et plus, afin de maintenir les objectifs à l’avenir.

## Introduction

Tetanus is a vaccine-preventable disease that causes significant avoidable morbidity and mortality globally. Infections are caused by wound contamination with bacterial *Clostridium tetani* spores, commonly found in dirt, soil, dust, and in the gastrointestinal tracts of many animals worldwide (Roper et al., 2007). Tetanus is characterized by intense muscle rigidity and spasms starting in the neck and face, often presenting as lockjaw (Roper et al., 2007). Unlike many other infectious diseases, tetanus cannot be transmitted from one individual to another (Roper et al., 2007). Therefore, widespread routine immunization is imperative for prevention, as herd immunity is not applicable (Roper et al., 2007).

Maternal and neonatal tetanus (MNT) are forms of generalized tetanus that occur during pregnancy or within 6 weeks after (maternal) and within 3–28 days after birth (neonatal) (World Health Organization [WHO], 2018a). In the 1980s, the World Health Organization (WHO) estimated over one million deaths each year were attributable to neonatal tetanus, resulting in a global mortality rate of 6.7 neonatal tetanus deaths per 1000 live births (Roper et al., 2007). Decades of concerted effort targeting vaccination coverage, birth hygiene, sterile umbilical cord care, and surveillance has successfully lowered MNT incidence rates worldwide (Thwaites et al., 2015; WHO, 2020a). By 2018, WHO estimated 25,000 deaths occurred due to neonatal tetanus, around a 97.5% increase from the 1980s (WHO, 2020b). WHO defines MNT elimination as the occurrence of less than one neonatal case per 1000 live births (WHO, 2018a, 2018b). MNT has been eliminated in the Region of the Americas in 2017 and in Europe in 2010, but remains a leading cause of preventable neonatal mortality in many African, Eastern Mediterranean, and Southeast Asian countries (Dikici, 2016; Pan American Health Organization [PAHO], 2017; WHO, 2020a).

To track national progress on vaccine-preventable diseases, Canada released a series of vaccination coverage goals and reduction targets to be achieved by 2025 (Public Health Agency of Canada [PHAC], 2021). The tetanus reduction targets outlined in the immunization strategy aim to maintain fewer than five cases of tetanus annually and maintain zero cases each of maternal and neonatal tetanus annually (PHAC, 2017, 2021). Although the incidence of tetanus has been and remains low, and MNT was eliminated well before 2000, Canada is currently unable to report zero cases of MNT due to the lack of specificity in the national case definition (PHAC, 2021; WHO, 2020a). The Canadian Notifiable Disease Surveillance System (CNDSS) does not differentiate MNT cases from other forms of tetanus, rendering Canada unable to assess both reduction targets by 2025 using CNDSS data alone (PHAC, 2008). Given the severity of tetanus, it is assumed that all cases require hospitalization. As such, this report aims to supplement CNDSS data with hospitalization and vital statistics data to appraise all cases of tetanus from 1995 to 2019 to describe the epidemiology and to assess progress on national reduction targets, including validating Canada’s status as having eliminated MNT.

## Methods

### Case definitions

The Canadian national case definition used for tetanus from 1995 to 2019 indicates a confirmed case as:

Clinical evidence of illness without other apparent medical cause with or without isolation of *Clostridium tetani* and with or without history of injury. Clinical illness is characterized by acute onset of hypertonia and/or painful muscular contractions (usually of the muscles of the jaw and neck), and generalized muscle spasms without other apparent medical cause (Health Canada, 2000; Health and Welfare Canada, 1991; PHAC, 2008).

For maternal tetanus, the WHO case definition is consistent with the general tetanus case definition with the stipulation that tetanus occurs during pregnancy or within 6 weeks after pregnancy ends (birth, miscarriage, or abortion) (WHO, 2018b).

WHO defines a confirmed neonatal tetanus case as:

[Case has] all three of the following: normal ability to suck and cry during the first two days of life AND could not suck normally between 3 and 28 days of age AND developed muscle stiffness and/or spasms (jerking) without an alternate diagnosis (WHO, 2018a).

### Data sources

#### Hospitalizations 

Tetanus hospitalizations with admission dates from January 1, 1995 through December 31, 2019 were obtained from the Canadian Institute for Health Information (CIHI) in May 2021. From 1995 to 2010, records were extracted from the Hospital Morbidity Database (HMDB) and 2011–2019 data were extracted from the Discharge Abstract Database (DAD). The DAD comprises records for all of the provinces and territories except Québec (Canadian Institute for Health Information [CIHI], 2021).

Cases were extracted using the ICD-10 codes *A33 Tetanus neonatorum*, *A34 Obstetrical tetanus*, or *A35 Other tetanus* as the primary diagnosis code. When ICD-10 information was unavailable, ICD-9 codes *037 Tetanus* or *771.3 Tetanus neonatorum* were used to filter data. Code *037 Tetanus* with an additional diagnosis of any code from the *Complications of Pregnancy, Childbirth, and the Puerperium* category (*630–679*) was used as a proxy for obstetrical tetanus in the ICD-9 system because no exact equivalent code exists.

Neonatal tetanus hospitalization records were excluded if the total length of stay was less than 3 days long to comply with the age requirement of the WHO case definition of neonatal tetanus (WHO, 2018a). Further, neonatal records were cross-referenced with provincial/territorial public health officials to confirm cases met the case definition.

#### Vital statistics 

Mortality data were extracted from Statistics Canada’s Canadian Vital Statistics Deaths Database (CVSD) in June 2021. Vital statistics data are unavailable for the provinces of Québec from 2001 to 2019, Ontario from 2016 to 2019, and Yukon from 2016 to 2019. ICD-9 and ICD-10 codes were used to filter cause of death information related to tetanus.

#### National case reports 

Nationally reported confirmed cases of tetanus from 1995 through 2019 were queried from the CNDSS in June 2021.

#### Population estimates

Demographic estimates from Statistics Canada were used as denominators to calculate appropriate tetanus incidence rates per 100,000 population and neonatal tetanus rates per 1000 live births (Statistics Canada, 2020, 2021). Québec populations were subtracted from 2011 to 2019 to reflect appropriate denominators for the available hospitalization data. For neonatal tetanus, Québec birth data were queried from the Institut de la statistique du Québec (Institut de la statistique du Québec, 2021).

### Descriptive and statistical analyses

Descriptive and statistical analyses for this report were conducted in R version 4.0.2 and Microsoft Excel 2016. Duplicate hospitalization records were removed by identifying unique health card numbers. In cases where the health card number was unavailable (occurring in fewer than 6% of records), deterministic record matching was used based on birth year, sex, province of occurrence, and postcode variables.

Statistical significance between categorical variables was assessed with chi-square and Fisher’s exact tests. Poisson regression and exact Poisson tests were used to estimate incidence rates. For length of hospital stay analyses, outliers that were more than three standard deviations away from the mean were excluded from calculations. To determine trends in seasonality, spring was categorized as a date of admission during the period March–May, summer June–August, autumn September–November, and winter December–February. For analyses of geographical distribution, the provinces and territories were divided into four regions: North, East, West, and Central (Table 1).

**Table 1:** Distribution of the Canadian provinces and territories into geographic regions

**Region**	**Included provinces and territories**
North	Yukon, Northwest Territories, and Nunavut
East	New Brunswick, Nova Scotia, Prince Edward Island, and Newfoundland and Labrador
West	British Columbia, Alberta, Saskatchewan, and Manitoba
Central	Ontario and Québec

## Results

### Hospitalizations

#### Non-MNT tetanus

**Fig. 1:**
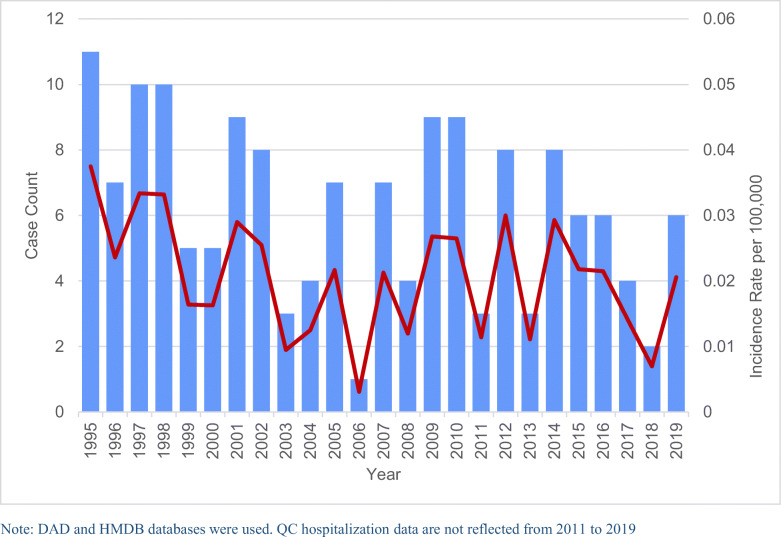
Tetanus cases and incidence rates in Canada, 1995–2019, reported from CIHI hospitalization data

From 1995 to 2019, there were 155 cases of tetanus (non-MNT) recorded from hospitals in Canada, corresponding to an overall incidence rate of 0.021 cases per 100,000 population (Table 2). From 1995 to 2006, the incidence rate decreased on average by 9.76% per year (*p*<0.01). Since 2006, the yearly incidence rates have not significantly changed. The average number of cases per year was 6.2 (range: 1–11) (Fig. 1). Seasonally, significantly fewer tetanus cases occurred in the winter compared to in all other seasons (*p*<0.01).

**Table 2:** Sex and regional distribution of tetanus incidence and fatalities in Canada, 1995–2019

	Tetanus cases	Incidence rate per 100,000 (95% CI)	Tetanus deaths^a,b^
Total	155	0.021 (0.017, 0.024)	10
Sex			
Male	88	0.024 (0.019, 0.029)	–
Female	67	0.018 (0.014, 0.022)	–
Region			
Central	78	0.018 (0.014, 0.022)	6
East	18	0.030 (0.018, 0.048)	–
West	59	0.023 (0.018, 0.030)	–
North	0	0	0

^a^Vital Statistics data exclude Québec 2001–2019, Ontario 2016–2019, and Yukon 2016–2019

^b^Some cells suppressed because of small cell sizes

Patients diagnosed with tetanus occupied an average of 72.8 hospital bed-days per year, excluding outliers (range: 5–449). The average length of an acute care hospital stay was 11.9 days, excluding outliers (range: 0 to 183). Most cases of tetanus (*n*=76; 49.0%) were discharged home after hospitalization without requiring further support. Others required transfer to another facility providing inpatient care (*n*=60; 38.7%), long-term care (*n*=7; 4.5%), medical support at home (*n*=4; 2.5%), or other (*n*=8; 5%).

Of all tetanus cases, 56.8% (*n*=88) were among males and 43.2% (*n*=67) were among females and this difference was statistically significant (*p*<0.05) (Table 2). The overall incidence rates for males (0.024 cases per 100,000) and females (0.018 cases per 100,000) were not significantly different (Table 2). However, when observing only cases less than 70 years of age, incidence rates were significantly higher for males (0.022 cases per 100,000) than for females (0.013 cases per 100,000) (*p*<0.01). Among those 70 years and older, the incidence rate was slightly higher for females than for males (*p*<0.05).

**Fig. 2:**
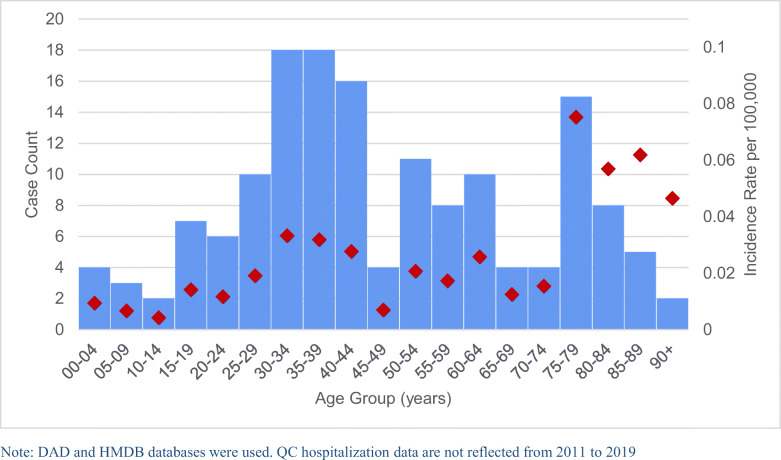
Tetanus cases by age group in Canada, 1995–2019, reported from CIHI hospitalization data

The average age of tetanus cases was 47.2 years (range: <1 year to 93 years). The age group with the highest overall incidence rate was 75–79 year-olds (0.075 cases per 100,000 population), and the age group with the lowest was 10–14 year-olds (0.004 cases per 100,000 population) (Figure 2). Incidence rates for those 75 and over were 3.87 times the rate for Canadians under 75 (*p*<0.001), controlling for year and sex.

The Central, East, West, and North regions of Canada accounted for 50% (*n*=78), 12% (*n*=18), 38% (*n*=59), and 0% (*n*=0) of tetanus cases over the 25-year period, respectively (Table 2).

From 1995 to 2019, a total of 10 deaths were recorded from tetanus (Table 2). The average age of death was 78.3 years (range: 43–93).

#### Maternal and neonatal tetanus

A total of 6 neonatal tetanus hospital records and 0 maternal tetanus records were retrieved from 1995 to 2019. After cross-referencing with provincial partners, all 6 cases were determined to be diagnostic coding errors or database entry errors, resulting in 0 cases included in the final analysis.

### National notifications

During the timeframe of interest, 91 cases of general tetanus were reported through the CNDSS, corresponding to an overall incidence rate of 0.011 cases per 100,000 population. Out of cases, 37% (*n*=34) were females and 63% (*n*=57) were males which was statistically significant (*p*<0.01). Geographically, 53% (*n*=48) of cases were located in the Central region, 7% (*n*=7) in the East, 40% (*n*=36) in the West, and 0% (*n*=0) in the North. However, the overall incidence rates for regions with cases were not significantly different. Further case breakdowns by age, sex, and year are available at:

https://diseases.canada.ca/notifiable/charts-list.

**Fig. 3:**
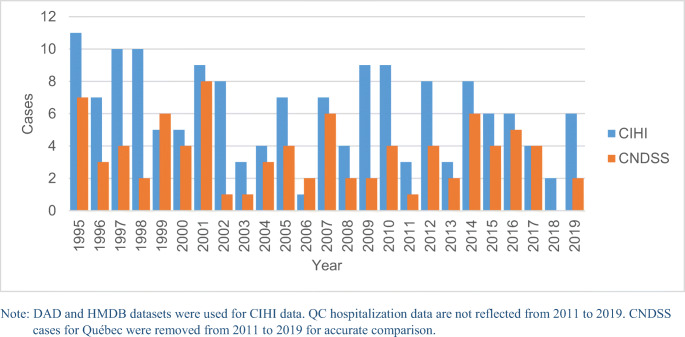
CNDSS national tetanus notifications and CIHI tetanus hospitalizations compared by year

CNDSS cases (*n*=87), excluding Québec cases from 2011 to 2019, only account for 56% of cases reported from CIHI (*n*=155) (Figure 3).

## Discussion

This paper supports the claim that MNT has been eliminated in Canada, finding zero cases of MNT in the study period. Although not possible to exclude the occurrence of MNT from 1995 to 2019, as errors do occur, the probability is extraordinarily low. Through consultation and validation with provincial/territorial public health officials, the 6 cases of neonatal tetanus originally queried from CIHI’s hospital database were all determined to be coding or database input errors. In the literature, errors have been reported among ICD-9 codes, such as incorrectly interchanging codes *773.1* and *771.8* with the neonatal tetanus code *771.3* (Boulianne et al., 2010).

Non-MNT case counts and incidence rates in Canada remain low, attributable to effective vaccination programs in Canada. In 2019, it was estimated that 78% of Canadian children received all recommended doses of a tetanus toxoid–containing vaccine by age two (PHAC, 2022). However, some provincial estimates with more efforts to validate vaccination with medical records found much higher coverage rates among 2 year-olds (Ji et al., 2022; Kiely et al., 2021). Overall, childhood immunization coverage appears sufficient in preventing significant disease burden from tetanus, although national estimates suggest Canada is still under the national goal of achieving 95% coverage by 2 years of age (PHAC, 2021). Seasonally, tetanus cases occur less often in the winter compared to in other seasons, when most people spend less time outside gardening or performing other activities in contact with materials containing *C. tetani* spores (Tiwari et al., 2020).

Adults 75 and older have significantly higher incidence rates of tetanus, which may be attributable to waning immunity over time. Tetanus booster shots are currently recommended to adults every 10 years to maintain immunity (PHAC, 2020). However, tetanus vaccination coverage among Canadian adults is traditionally lower than in childhood and declines as age increases (PHAC, 2019). Declining tetanus vaccination coverage with old age is also observed in countries similar to Canada, including Switzerland and Australia (Bovier et al., 2001; Quinn & McIntyre, 2007). It is unclear how older adults are getting exposed to the bacteria. Increasing individual protection through vaccination is the best method to prevent tetanus.

The results provide some evidence that statistically more cases occurred in males than in females among hospitalizations and national notifications, particularly for those under 70 years of age. Higher incidence rates of tetanus among males as compared to females have been previously reported in the literature, although not consistently (Kyu et al., 2017; Marulappa et al., 2012). Differential occupational exposure to *C. tetani* bacteria in outdoor and fieldwork may explain why men are disproportionately affected by tetanus among working age groups (Marulappa et al., 2012).

This report affirms that tetanus deaths are rare in Canada. The high average age of death indicates that fatalities most often occur among older adults, which is consistent with the current literature (Kyu et al., 2017).

An unexpected finding from this report is the lack of tetanus hospitalizations in the North region. There is evidence that *C*. *tetani* bacteria are more prevalent in densely populated areas with hot moist climates, which may explain the lack of cases in cool and sparsely populated northern regions (Tiwari et al., 2020). Further, due to the small population size living in the North region, it is feasible that no tetanus cases occurred during this time. However, it is also possible that true cases of tetanus were missed as a result of diagnostic coding errors.

The last notable finding was the discrepancy between national notifications for tetanus and the number of cases in Canadian hospitals. Given that each case of tetanus is assumed to be hospitalized, CNDSS notifications and cases from hospitalizations are expected to be similar, although this was not observed. Diagnostic coding errors in the DAD and HMDB databases could contribute to higher tetanus hospitalization counts and overestimate the true number of cases, although this is less likely to occur within the primary diagnosis field used in this report (CIHI, 2016). This could also indicate that hospitalizations for tetanus are not being captured at the provincial level and are subsequently not reported in federal estimates.

### Assessment of VPD targets

The Canadian vaccine-preventable disease reduction targets by 2025 for tetanus are outlined in Table 3. The first target aims to maintain fewer than five cases of tetanus annually. Tetanus case counts per year remain low and average yearly cases are decreasing. In recent years (2016 through 2019), Canada is on track to meet the target (Table 3). Canada has successfully achieved the targets for MNT during the entire period of study (Table 3).

**Table 3:** Canada’s progress towards the tetanus vaccine–preventable disease reduction targets by 2025

Target	1995–2010 average yearly cases (range)	2011–2015 average yearly cases (range)	2016–2019 average yearly cases (range)
Maintain less than five cases of tetanus annually in Canada	7.06 (1–11)	5.80 (3–8)	4.50 (2–6)
Maintain zero cases of neonatal tetanus in Canada	0	0	0
Maintain zero cases of maternal tetanus in Canada	0	0	0

### Limitations

Hospitalization data in this report are likely underestimated. This is primarily because Québec hospitalizations are unavailable from 2011 to 2019. CIHI states that Québec data typically represent 25% of all hospital separations each year (CIHI, 2009). However, the online hospitalization database *Maintenance et exploitation des données pour l’étude de la clientèle hospitalière *(MED-ÉCHO) indicates there were only 7 hospitalizations in Québec with tetanus as the primary diagnosis code from April 1, 2010 to March 31, 2020 (Ministère de la Santé et des Services sociaux, 2021). Hence, it is estimated that approximately 13% of hospitalizations are not included in this report from 2011 onwards. The MED-ÉCHO and CIHI databases were not able to be merged. In addition, tetanus requires a high index of suspicion to make a correct diagnosis given how rare it is. Consequently, some cases may have been missed across all provinces. It is also possible that some valid tetanus cases had ICD codes in additional diagnosis fields 2-25, and would therefore not meet the inclusion criteria for this report. CIHI diagnosis codes for non-MNT tetanus were not validated in this study. This means some uncertainties about the specificity of the diagnoses remain.

A further limitation is the small annual case counts associated with low incidence rates of tetanus. The low number of cases can result in unstable rates over time, as a single case can alter the incidence rate considerably. Associated inference and confidence intervals should be interpreted with caution based on low count numbers. Further, due to the small number of fatalities, this report is unable to make conclusions about the demographics of fatal cases.

A future study could perform a case review of the non-MNT cases to validate hospital codes with original healthcare records. This could increase the confidence in true case counts and help understand the discrepancy between CIHI and CNDSS case counts. Subsequent reports can consider adding the history of injury and vaccination status when studying tetanus. History of injury may provide insight for clinicians on tetanus diagnosis based on information from previously confirmed cases. Similarly, documenting rare cases with no evidence of injury may help identify comparable cases in the future with improved accuracy. From a public health management perspective, vaccine status is a valuable metric to help identify gaps in immunization efforts and inform programs.

## Conclusion

This report provides a comprehensive look at the epidemiology of tetanus in Canada from 1995 to 2019. As anticipated, the incidence of tetanus remains low, with the highest rates occurring in older adults. Annual case counts are low and in recent years Canada has achieved the reduction target of maintaining fewer than 5 cases per year. Remaining vigilant with routine vaccinations for all age groups, including those aged 75 and older, is required to sustain this target in upcoming years. In addition, this is the first paper to provide previously unavailable case information for maternal and neonatal tetanus in Canada’s recent history. This report affirms that Canada has achieved elimination status of MNT and has accomplished the national targets of reporting zero cases of maternal and of neonatal tetanus annually from 1995 to 2019. Understanding tetanus epidemiology through sustained surveillance efforts will aid public health management and allow ongoing assessments for the reduction targets to mitigate disease burden in Canada.

## Contributions to knowledge

What does this study add to existing knowledge?

- To the best of our knowledge, this is the first study in Canada to use hospitalization data to validate Canada’s claim as having eliminated maternal and neonatal tetanus.
- The study provides an updated report on the national epidemiology of tetanus in Canada using national notification, hospitalization, and death data.
- Adults 75 and older were observed to have incidence rates of tetanus 3.87 times higher than Canadians under 75, controlling for year and sex.

What are the key implications for public health interventions, practice or policy?

- Routine childhood immunization coverage in Canada appears sufficient in preventing significant disease burden from tetanus, as incidence rates are quite low.
- Remaining vigilant with routine vaccinations, including for adults 75 and over, is required to reach and sustain Canadian tetanus reduction targets in upcoming years.
- Discrepancies in tetanus case counts between national notifications and hospitalizations could mean tetanus cases are not being captured at the provincial level and are subsequently not reported nationally.

## Data Availability

The data sources used in the study are a mix of openly available data (including nationally reported confirmed tetanus cases from the Canadian Notifiable Disease Surveillance System) and private data from Statistics Canada (mortality data) and the Canadian Institute for Health Information (hospitalization data). As such, restrictions apply to the availability of private data, which were used in this study under data-sharing agreements.
